# Management of progressive joint disorders by non-physician healthcare providers in South Africa: A mapping study to inform pharmacist integration

**DOI:** 10.4102/phcfm.v18i1.5281

**Published:** 2026-05-11

**Authors:** Tumelo Modau, Demitri Constantinou, Ané Orchard

**Affiliations:** 1 Department of Pharmacy and Pharmacology, Faculty of Health Sciences, University of the Witwatersrand, Johannesburg, South Africa; 2 Department of Exercise Science and Sports Medicine, Faculty of Health Sciences, University of the Witwatersrand, Johannesburg, South Africa

**Keywords:** allied health professionals, interprofessional collaboration, musculoskeletal, pharmacist integration, referral pathways, rehabilitative healthcare providers

## Abstract

**Background:**

Progressive joint disorders (PJDs), including osteoarthritis, rheumatoid arthritis (RA) and are leading causes of disability worldwide. Early multidisciplinary intervention is critical; however, delayed non-pharmacological management, fragmented referral pathways and limited integration of pharmacists, despite their potential role in medication management and early ‘red flag’ identification, remain common.

**Aim:**

To identify which PJDs are managed by which healthcare providers, to inform pharmacist referral pathways and promote integrated care.

**Setting:**

Non-physician healthcare providers (HCPs) in South Africa, including physiotherapists, occupational therapists, chiropractors, podiatrists, osteopaths, biokineticists and other complementary therapy providers.

**Methods:**

A quantitative, cross-sectional survey was administered via REDCap^®^ and distributed by professional bodies through email and professional association platform advertisements. Descriptive statistics and quantitative content analysis assessed condition management, referral practices and collaboration.

**Results:**

A total of 266 HCPs participated. Osteoarthritis (91.7%) and RA (84.6%) were the most managed conditions. High interprofessional collaboration was noted with physiotherapists (71.4%) and limited with pharmacists (23.3%). Most providers (76.3%) did not require referral letters. Referrals to and from other providers were common, but incoming referrals varied by profession. Internal consistency was acceptable (Cronbach’s α > 0.70).

**Conclusion:**

Progressive joint disorders were predominantly managed by chiropractors, physiotherapists and biokineticists. Referral pathways were inconsistent, and pharmacist integration remained limited.

**Contribution:**

This study presents the first national mapping of non-physician roles in PJD management in South Africa, providing critical insights for future development of pharmacist-inclusive referral guidelines.

## Introduction

Progressive joint disorders (PJDs) are a group of chronic musculoskeletal conditions characterised by the gradual deterioration of joint structures because of degenerative, inflammatory or metabolic processes.^1^ These conditions include osteoarthritis, rheumatoid arthritis, gout, chronic low back pain, spondylitis and psoriatic arthritis, among others.^2,3^ Progressive joint diseases impair mobility, limit daily functioning, reduce quality of life and impose a significant economic burden on healthcare systems because of prolonged treatment and hospitalisation, loss of productivity and the necessity for complex interventions such as arthroplasty.^3^

Early identification and management of these conditions are crucial for slowing disease progression and preventing irreversible joint damage.^4^ Timely intervention can improve pain control, maintain joint function and significantly enhance patient outcomes.^4^ However, despite the availability of effective non-pharmacological strategies, early intervention is often delayed or overlooked in clinical settings.^4,5^ Research shows that physicians frequently prioritise pharmacological management, with 52.3% of rheumatologists indicating a preference for medication as a stand-alone over referring patients to physiotherapy.^5^ In conditions such as osteoarthritis, referrals to non-physician healthcare providers are often delayed until late-stage disease, resulting in missed opportunities for early intervention, which could delay the need for surgery by 5 years to 7 years.^6^

Effective management of PJDs relies on a combination of pharmacological and non-pharmacological approaches.^2^ Pharmacological strategies can alleviate symptoms and slow disease progression; however, they are often insufficient as standalone therapies.^2,4^ Non-pharmacological interventions, delivered by non-physician healthcare providers, address critical aspects of patient care, including pain reduction, functional restoration and improved quality of life.^7^ Non-physician healthcare providers such as physiotherapists tailor mobility programmes, podiatrists manage rheumatoid arthritis-related foot issues, occupational therapists provide ergonomic solutions, biokineticists focus on exercise-based rehabilitation, and dieticians support disease management through nutritional interventions aimed at weight management, inflammation reduction and metabolic control.^8,9,10,11^ Given the chronic, complex nature of PJDs, with varying management strategies depending on disease stage, and other patient-related factors, a multidisciplinary, patient-centred approach is essential; yet non-physician healthcare providers remain underutilised or are engaged too late in the disease trajectory.^2,6,11^

Pharmacists are highly accessible healthcare providers with expertise in medication management and are well-positioned to contribute proactively to musculoskeletal care by identifying clinical red flags such as persistent joint pain, gait abnormalities, unresponsive symptoms or inadequate response to pharmacological therapies and initiating timely referrals and supporting early intervention.^12,13^ Despite this potential, pharmacists remain underutilised in musculoskeletal care because of barriers including limited interprofessional communication, unclear referral protocols, insufficient role clarity, gaps in training and confidence, and structural constraints such as inadequate institutional support, remuneration models and shared health information systems.^14,15,16^

Addressing these gaps is particularly important within the context of South Africa’s National Health Insurance (NHI), where coordinated referral systems and integrated care pathways are ‘being mooted as’ central to improving equitable access, reducing the burden of chronic conditions, such as PJDs, and enhancing continuity of care across the healthcare system.^17^

The purpose of this study was to identify which PJDs are managed by which healthcare providers in South Africa, with the aim of informing the future development of referral guidelines for pharmacists. By mapping provider-specific involvement in conditions such as osteoarthritis, rheumatoid arthritis, gout, ankylosing spondylitis and psoriatic arthritis, the study aims to support more effective interprofessional collaboration, which includes pharmacists, ensuring timely and appropriate referrals, and ultimately improving patient outcomes through integrated musculoskeletal care.

## Research methods and design

### Study design

The study adopted a quantitative, cross-sectional descriptive design, incorporating a survey with both closed and open-ended questions.

### Study setting and population

The study was conducted across South Africa and included rehabilitative or non-medical healthcare providers and complementary and alternative medicine (CAM) providers actively involved in the management of PJDs. For the purposes of this study, these groups are collectively referred to as ‘non-physician healthcare providers’. This category comprises physiotherapists, occupational therapists, chiropractors, podiatrists, osteopaths, biokineticists, acupuncturists and others (e.g. reflexologists, sports massage therapists, homeopaths). These participants were recruited nationwide and represented both private and public sector healthcare settings. Healthcare providers who exclusively manage pre-adolescent patients were excluded from the study, as they are unlikely to encounter or manage some of the PJDs under investigation. Data collection occurred between 01 May 2024 and 31 March 2025.

### Sampling method

A non-probability purposive sampling approach was employed to ensure the inclusion of a broad and diverse range of healthcare professionals actively involved in musculoskeletal care. Potential participants were identified through multiple recruitment channels, including approaching professional regulatory bodies and professional associations. These organisations distributed the survey link directly via email to their registered healthcare professionals or posted it on their official communication platforms, including websites and social media pages, allowing members to voluntarily access the survey.

### Sample size determination

A total of 266 valid responses were included in the final analysis. A formal a priori sample size calculation was not performed; however, the sample size was deemed adequate for descriptive analysis and exploratory comparisons across provider subgroups, consistent with the study’s exploratory and mapping objectives.

### Questionnaire development

The questionnaire was developed using the Research Electronic Data Capture (REDCap^®^) platform hosted by the University of the Witwatersrand, version 14.8.3, and included sections on demographics, PJDs managed, medicinal or supportive product use, referral practices and interprofessional collaboration. Prior to completing the questionnaire (Appendix 2), participants were required to read a study information sheet and provide informed consent electronically by signing the participant consent sheet (Appendix 1).

### Pilot testing

The questionnaire was pilot-tested with a subset of eight non-physician healthcare providers from different disciplines, who were randomly selected and excluded from the final analysis. Feedback from the pilot led to improvements in question clarity, logic flow and technical functionality.

### Reliability testing

The internal consistency of key multi-item constructs, such as collaboration and referral practices, was assessed using Cronbach’s alpha coefficient (α), with a value of ≥ 0.70 considered acceptable.

### Sampling and data collection procedure

A non-probability purposive sampling approach was employed to ensure the inclusion of a broad and diverse range of healthcare professionals actively involved in musculoskeletal care. Data were collected online. Potential participants were identified through multiple recruitment channels, including approaching professional regulatory bodies (Allied Health Professions Council of South Africa [AHPCSA] and Health Professions Council of South Africa [HPCSA]) and professional associations (Occupational Therapy Association of South Africa, Podiatry Association of South Africa, Physiotherapy Association of South Africa, Biokinetics Association of South Africa, Chiropractic Association of South Africa, Osteopathic Association of South Africa and Acupuncture Association of South Africa), which posted the survey link on their social media platforms (Facebook and LinkedIn pages) or distributed it directly via email to their registered healthcare professionals, allowing members to voluntarily and anonymously access the survey. The links were re-posted every 6 weeks post-launch to improve response rates.

### Data management and analysis

Closed-ended data were exported from REDCap^®^ onto STATA^®^ SE 18 statistical software, employing descriptive statistics including frequencies, percentages, means, medians, standard deviations and Cronbach’s alpha. Open-ended responses were analysed using qualitative content analysis to identify referral-related themes and were managed using Microsoft^®^ Excel^®^ version 2411 and Maximum Qualitative Data Analysis (MAXQDA) version 24.9 software to ensure systematic coding and verification.

### Ethical considerations

Ethical clearance to conduct this study was obtained from the University of the Witwatersrand Human Research Ethics Committee (No. M220344). All participants provided informed consent electronically, and data were stored securely with no collection of personally identifiable information.

## Results

A total of 266 participants completed the survey (Table 1-A3 Appendix 3), and 192 participants (73.3%) were female. The participants were predominantly white people, with 190 (71.4%) participants, while other racial groups were less represented. Ninety-five participants (35.7%) were aged between 30 years and 39 years, while participants over 60 years constituted the smallest proportion (7.1%).

**FIGURE 1:**
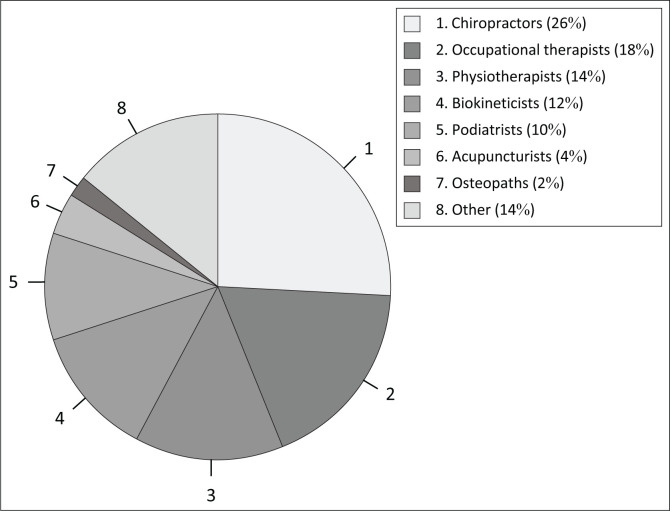
Healthcare providers who participated in the survey.

The healthcare providers who participated in the study are shown in Figure 1, where 69 (25.9%) were chiropractors, who constituted the majority. The least represented were osteopaths, with six (2.3%) participants. Other healthcare providers accounted for 13.9%, including homeopaths, sports massage therapists, phytotherapists, reflexologists and aromatherapists.

**FIGURE 2:**
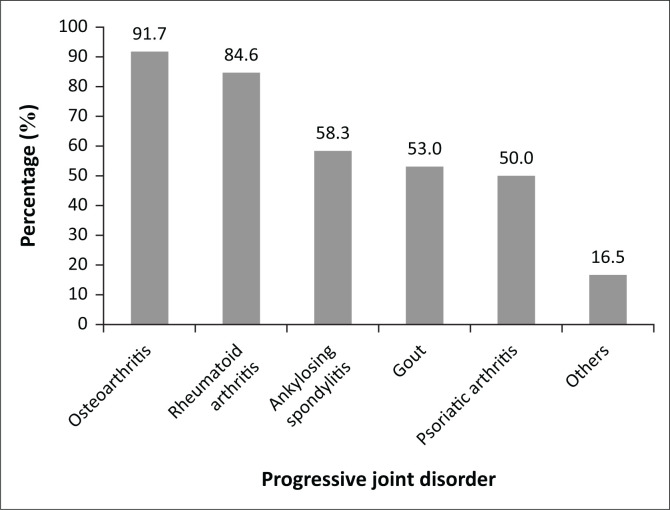
Most commonly managed progressive joint disorders.

Figure 2 illustrates the most commonly managed PJDs, with osteoarthritis reported by 244 participants (91.7%). In contrast, psoriatic arthritis was the least frequently reported, with only 133 participants (50.0%) indicating its management. ‘Other’ PJDs were mentioned by 44 participants (16.5%), citing conditions such as spondylolisthesis, various muscle, ligament, tendon, and joint pathologies, sports injuries, spine or back and neck-related disorders, fibromyalgia and scoliosis, among others. A complete list of ‘other’ PJDs is available in Table 1-A3 Appendix 3. The reliability analysis showed a Cronbach’s alpha of 0.85 for the top five conditions, indicating a high level of internal consistency among the items.

Details on which healthcare provider manages which PJDs were reported (Table 1-A3 Appendix 3). Osteoarthritis was the most commonly managed condition, reported by 32 biokineticists (100.0%), 10 acupuncturists (100.0%), 6 osteopaths (100.0%), 67 chiropractors (97.1%) and 26 podiatrists (96.3%). Across all providers, the percentages of those managing osteoarthritis yielded a mean of 93.8% and a standard deviation (s.d.) of 9.7%. The median percentage was 96.7%. The interquartile range (IQR) was 6.1%, with the first quartile (Q1) at 93.9% and the third quartile (Q3) at 100.0%. The highest reported management of rheumatoid arthritis was among podiatrists and osteopaths, both at 100.0% (27 and 6 participants, respectively), with the statistical mean across all providers being 87.9% (s.d. = 9.7%). Psoriatic arthritis was reported to be managed by only 10 physiotherapists (27.0%), 12 occupational therapists (25.0%) and 3 acupuncturists (30.0%). Similarly, gout had lower reporting rates among occupational therapists, with 13 participants (27.1%) and 10 biokineticists (31.3%) reporting managing it. In contrast, ankylosing spondylitis was relatively less reported among occupational therapists, with 15 participants (31.3%), and podiatrists, where only 11 participants (40.7%) indicated managing this condition.

**FIGURE 3:**
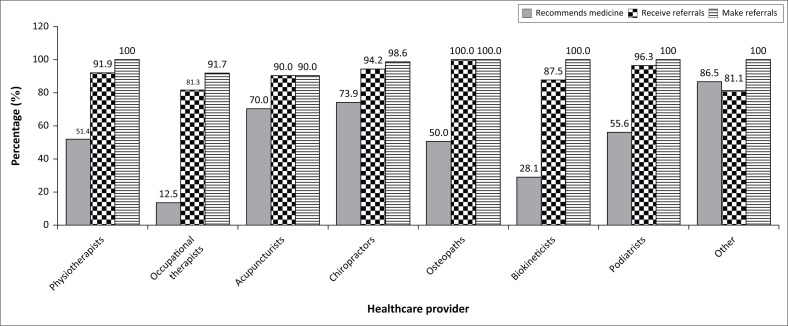
Medicinal recommendations and referrals by healthcare providers.

Figure 3 illustrates the referrals and medicinal recommendations by healthcare providers. All (100%) physiotherapists, osteopaths, biokineticists, podiatrists and other healthcare professionals reported that they refer patients to other providers. Occupational therapists reported the lowest rate of medicinal recommendations, with only six participants (12.5%) indicating they do so. Additionally, all six osteopaths stated that they receive their patients through referrals.

**FIGURE 4:**
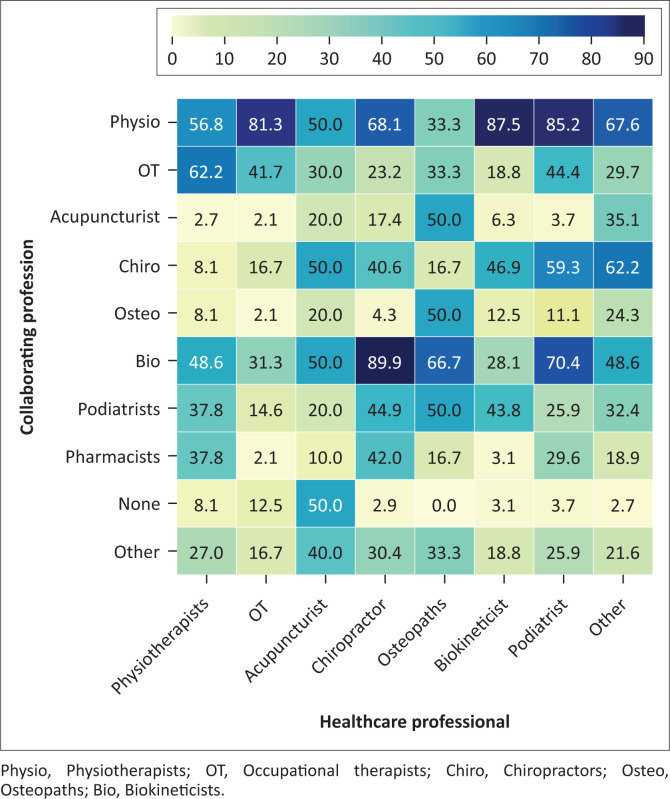
Cross-disciplinary collaboration matrix among non-physician healthcare providers (%).

The patterns of collaboration among healthcare providers were further explored (Figure 4). Of the participants, 62 chiropractors (89.9%) and 19 podiatrists (70.4%) reported collaborating predominantly with biokineticists. Overall, 190 participants (71.4%) indicated collaboration with physiotherapists, whereas 62 participants (23.3%) reported collaborating with pharmacists. Collaboration with pharmacists was notably limited, with only one occupational therapist (2.1%), one acupuncturist (10.0%), one osteopath (16.7%) and one biokineticist (3.1%) reporting such interaction. The nine-item binary scale demonstrated acceptable internal consistency, with a Cronbach’s alpha of 0.78, indicating that the items reliably captured patterns of collaboration between different healthcare providers and pharmacists.

**FIGURE 5:**
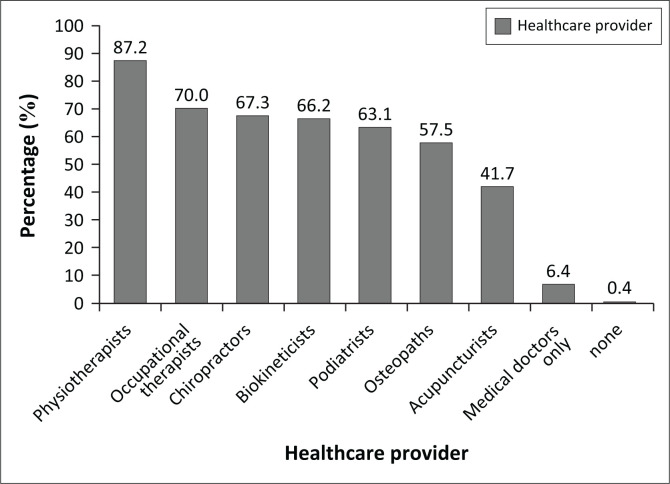
Healthcare providers to collaborate with pharmacists.

Figure 5 illustrates the percentage of healthcare providers who reported collaboration with pharmacists. A total of 232 healthcare providers (87.2%) indicated that pharmacists should collaborate with physiotherapists. The average collaboration rate across all healthcare provider categories was approximately 51.1%. Seventeen healthcare providers (6.4%) stated that pharmacists should collaborate exclusively with medical doctors, while only one participant (0.4%) reported that pharmacists should not collaborate with any healthcare provider. The seven-item binary scale (coded as 1 = yes, 0 = no) demonstrated good internal consistency, with a Cronbach’s alpha of 0.82, indicating strong reliability in capturing healthcare providers’ views on pharmacist collaboration with various healthcare providers.

In an open-ended question, participants were asked to describe the type of patients whom pharmacists should refer to them. The content analysis revealed several recurring categories related to referral needs, as shown in Table 1. The most frequently reported theme was pain-related conditions, particularly joint pain, chronic musculoskeletal discomfort and symptoms unresponsive to medication, as reported by 115 participants.

**TABLE 1:** Themes identified for pharmacist-initiated referrals to non-physician healthcare providers.

Referral theme	Description	Frequency (*n*)
Pain-related conditions	Joint pain, chronic musculoskeletal discomfort and symptoms unresponsive to medication	115
Mobility impairments	Gait abnormalities, balance problems, postural instability and movement limitations	41
Reduced quality of life	Functional limitation and reduced participation in daily activities because of musculoskeletal symptoms	34
Preference for non-pharmacological management	Desire to reduce long-term medication use and pursue physical or rehabilitative interventions	30
Rehabilitation needs	Need for structured rehabilitation, recovery or functional reconditioning	26
Splinting and orthotic support	Requirement for splints, braces or orthotic devices	12
Mental health considerations	Anxiety, psychosomatic factors or psychological contributors to musculoskeletal complaints	9

Lastly, 203 participants (76.3%) reported that they do not require a referral letter from patients. In contrast, 30 occupational therapists (60.4%) indicated that they do require a referral letter, as reported in Table 1-A3 Appendix 3.

## Discussion

The predominance of female healthcare providers in the study reflects well-documented workforce trends within South Africa and globally, where allied healthcare professions are largely female-dominated.^18,19,20^ The racial distribution aligns with previous findings that historical inequalities have continued to influence the racial composition of many professional groups.^20^ The relatively young and early-career profile of participants is consistent with international studies and national workforce planning priorities emphasising the development of younger healthcare cadres.^21^

The study participants were non-physician healthcare professionals who provide diagnostic, therapeutic and preventive services distinct from those provided by medical doctors, nurses or pharmacists.^22,23^ The study reveals a notable dominance of chiropractors, who constituted 25.9% of the participants. This aligns with statistics from the AHPCSA, which show that the number of chiropractors was the highest (*n* = 993) compared to other healthcare providers in this study: homeopaths (*n* = 600), acupuncturists (including Chinese Medicine and Acupuncture) (*n* = 232), phytotherapists (*n* = 50) and osteopaths (*n* = 28) registered with AHPCSA as of 02 May 2025.^22^ This finding further suggests that chiropractors may be more actively engaged in multidisciplinary research or healthcare networks than other physical therapy-related professions in South Africa. The osteopaths represented the smallest participant group, comprising six individuals (2.3%). This finding aligns with expectations, provided that, within the context of this research study, osteopaths constitute the least populous category within the AHPCSA registers, with only 28 registered practitioners documented as of 02 May 2025.^22^

According to the HPCSA registers, when examining the healthcare providers for this study, physiotherapists had the largest number of registered practitioners at 8783 as of 31 October 2024, followed by occupational therapists with 6233.^23^ However, this was not reflected in our study, as physiotherapists accounted for 13.9% of participants, ranking third, while occupational therapists ranked second with 18.1%. Although physiotherapists make up a large proportion of the national registry, they were underrepresented in this study. This may reflect differences in availability, workload or the perceived relevance of the study in daily practice. Similar disparities have been reported in other research, where survey participation did not align with registry data.^21^ This often relates to practice-setting differences, as practitioners in independent or community-based settings may have less oversight or encouragement to participate in research than those in hospital environments.^21^ Additional factors, such as a lack of incentives or the recruitment methods used, may also have influenced participation.^21^ It is therefore essential to interpret the study’s findings within the context of these potential limitations and to acknowledge that registry data alone may not accurately predict trends in healthcare research participation.

Healthcare providers’ management of PJDs demonstrated strong internal consistency (Cronbach’s α = 0.85). Osteoarthritis (91.7%) and rheumatoid arthritis (84.6%) were the most commonly managed conditions, reflecting global epidemiological trends that identify osteoarthritis as the most common form of arthritis worldwide, driven by an ageing population, obesity and the widespread exposure to joint injuries and biomechanical stresses.^24^ Nearly all providers reported involvement in the management of osteoarthritis, although occupational therapists were less involved (70.8%), highlighting a contrast between high condition prevalence and persistently low interprofessional collaboration reported in literature (30.0%), largely attributed to role ambiguity.^25^ Rheumatoid arthritis was managed by most providers (mean = 87.9%; s.d. = 9.7%), with podiatrists and osteopaths universally involved, consistent with evidence supporting podiatric management of foot complications and the use of osteopathic manipulative medicine as a supportive, non-pharmacological adjunct to conventional care.^26,27^

Ankylosing spondylitis (58.3%) and gout (53.0%) demonstrated a greater variation in provider involvement. Physiotherapists, acupuncturists, chiropractors and osteopaths were most involved in the management of ankylosing spondylitis, consistent with physiotherapy’s central role in maintaining spinal mobility and the use of adjunct modalities for symptom relief, despite limited long-term evidence and standardised protocols.^28,29^ Chiropractic and osteopathic involvement was higher than reported in some international studies, although literature emphasises cautious application of manual therapies in advanced disease because of structural spinal risks.^30,31^ Gout management was most frequently reported by podiatrists, osteopaths, acupuncturists and other complementary healthcare providers, aligning with evidence highlighting these providers’ role in managing foot-related manifestations.^8,32^ Conversely, the lower involvement of occupational therapists and biokineticists may reflect both limited referral pathways and scope-of-practice restrictions, suggesting underutilisation despite potential benefits in chronic disease management.^33,34^

Exactly half of the surveyed healthcare providers reported management of psoriatic arthritis, with the highest engagement observed among podiatrists, osteopaths, chiropractors and other complementary healthcare providers, reflecting the condition’s musculoskeletal burden in the hands, feet and spine.^35^ Lower involvement was observed among biokineticists, occupational therapists and physiotherapists, which may reflect limited exposure to inflammatory arthropathies in these roles or inconsistent referral practices.^36^ Despite the high burden of functional impairment associated with psoriatic arthritis, other healthcare providers remain underutilised in its management, underscoring the need for greater interdisciplinary collaboration and targeted training.^37^

More than half (53.4%) of healthcare providers reported recommending medicinal items. The highest rates of recommendation were observed among chiropractors, acupuncturists and those in the ‘other’ category, reflecting the increasing integration of pharmacological and complementary products within these practices.^38,39^ Conversely, occupational therapists and biokineticists exhibited the lowest frequency of medicinal item use, likely attributable to scope-of-practice restrictions and a principal focus on functional and physical rehabilitation.^34,40^ Physiotherapists and podiatrists presented moderate levels of medicinal item recommendations, aligning with their responsibilities in managing musculoskeletal and foot-related conditions, where adjunct medicinal support may be deemed necessary.^41,42^

The diverse patterns emphasised herein illustrate the critical necessity for interdisciplinary collaboration, particularly involving pharmacists, to ensure safe, evidence-based guidance in the use of medicinal products. Pharmacists possess specialised expertise in pharmacotherapy, drug interactions and patient education, rendering them essential collaborators in optimising therapeutic outcomes and facilitating informed prescribing or recommendation practices among non-physician healthcare providers.^43,44^ Emerging strategies, including the implementation of artificial intelligence-powered pharmacovigilance systems for detecting herb–drug interactions, may support safer practice, but require robust collaboration between pharmacists and non-physician healthcare providers.^45^

The majority of healthcare providers surveyed reported both referring to (97.7%) and receiving referrals from (89.1%) other healthcare providers, indicating a high degree of interprofessional collaboration. Osteopaths, podiatrists and chiropractors received the most referrals, and this aligns with findings from other studies, while occupational therapists and providers in the ‘other’ category received fewer referrals, reflecting under-recognition of their roles.^46,47,48^ These patterns align with prior evidence of persistent gaps in interdisciplinary communication and structural barriers to team-based care in certain healthcare settings.^49^ These findings emphasise the need to strengthen bidirectional referral pathways and promote broader awareness of each provider’s scope and contribution to patient care.

Collaboration was most frequently noted with physiotherapists, followed by biokineticists and chiropractors, reinforcing physiotherapists’ recognised central role in multidisciplinary musculoskeletal care.^42,50^ Lower collaboration with osteopaths and acupuncturists may reflect limited formal integration within conventional healthcare systems.^27^ Notably, occupational therapists reported the highest collaboration with physiotherapists, while podiatrists demonstrated the highest collaboration with biokineticists and chiropractors. These findings align with the literature recognising physiotherapists as central figures in multidisciplinary teams, particularly in musculoskeletal and rehabilitation settings. Conversely, the lower collaboration rates with osteopaths and acupuncturists may reflect a lack of integration or formal referral pathways in conventional healthcare systems. Only 7.1% of respondents indicated no collaboration, and the collaboration scale demonstrated acceptable reliability (Cronbach’s α = 0.78).

Despite the overall collaboration, interaction with pharmacists was limited (23.3%), echoing previous findings that unclear referral pathways and role ambiguity hinder pharmacist integration.^16,51^ This is concerning given the vital role pharmacists play in medication management, patient education and reducing adverse drug reactions and interactions.^52^ This is particularly important as many patients with joint diseases may be prescribed medicines whose side effects can directly impact therapy outcomes; for example, long-term corticosteroid use (e.g. cortisone) can weaken bones, impair cartilage integrity and increase fracture risk, while certain antiepileptic drugs may cause muscle weakness, reduce bone mineral density and impair balance, thereby raising the likelihood of falls and further injury.^53,54^ Chiropractors and physiotherapists reported the highest pharmacist collaboration, while occupational therapists and biokineticists reported the lowest, highlighting missed opportunities for integrated pharmacological and non-pharmacological care.^44,52^

Most providers supported pharmacist involvement in multidisciplinary care, particularly collaboration with physiotherapists, occupational therapists, chiropractors and biokineticists, and recommended that pharmacists refer patients with joint pain, musculoskeletal discomfort and symptoms unresponsive to medication. Broad acceptance of interdisciplinary collaboration was evident, with minimal support for pharmacist-only collaboration with medical doctors and strong internal consistency of this construct (Cronbach’s α = 0.82). Support for pharmacist collaboration was lowest with acupuncturists and osteopaths, likely reflecting philosophies and evidence bases; however, given patients’ frequent use of multiple care modalities, pharmacist awareness of complementary therapies remains essential for medication safety and coordinated care.^51,55^

Lastly, more than three-quarters of healthcare providers surveyed indicated that they do not require a referral letter for patient consultations, highlighting a high degree of accessibility across professions. Chiropractors, osteopaths, podiatrists and providers in the ‘other’ category reported the highest levels of openness to direct patient access. Increasing recognition of direct and pharmacist-initiated referrals highlights a shift towards more accessible, patient-centred care models, although improved standardisation and communication remain necessary to support integrated musculoskeletal care.^56^

### Limitations

The study has limitations, including the use of non-probability purposive sampling and relatively small sample size, compared with the total number of registered healthcare providers, which further limits representativeness. In addition, because the survey link was made publicly available through professional platforms, the exact number of healthcare providers approached could not be determined, nor could the geographical distribution of respondents be precisely assessed beyond confirming that participants were practising healthcare providers in South Africa. Reliance on self-reported data may be subject to response bias, and the cross-sectional design restricts causal inferences. Furthermore, dieticians and psychologists, who contribute to joint disease management through nutritional and psychosocial interventions, were not included, which may have limited the representation of multidisciplinary care pathways.

### Recommendations

- Develop and embed pharmacist-inclusive, evidence-based referral guidelines: To clarify provider roles, reduce inconsistent referral patterns and improve coordinated multidisciplinary PJD care. These innovations are particularly important within the framework of South Africa’s NHI, where integrated, efficient and equitable care pathways will be essential to meet the growing burden of chronic musculoskeletal conditions. Therefore, policies should ensure that pharmacist-inclusive pathways are embedded into NHI rollout protocols.
- Strengthen interprofessional education and role awareness: Through higher education institutions and professional bodies to address limited collaboration and underutilisation of pharmacists and other non-physician healthcare providers (HCPs).
- Expand future referral frameworks to include dieticians and psychologists: Acknowledging their roles in nutritional, behavioural and psychosocial management of joint disease not captured in this study.
- Leverage digital and artificial intelligence-enabled tools to enhance pharmacovigilance and support safe collaboration with CAM providers, particularly through the detection of herb–drug interactions.

## Conclusion

This study highlights that, while non-physician healthcare providers play a central role in the management of PJDs in South Africa, referral pathways remain inconsistent, and pharmacist involvement is limited despite their importance in medication management and early ‘red flag’ identification. Care is predominantly delivered by physiotherapists, biokineticists and chiropractors, with other providers underutilised, pointing to missed opportunities for coordinated multidisciplinary care. Strengthening pharmacist integration, clarifying referral roles and addressing collaboration gaps through targeted training and system-level reforms are essential to improve early intervention, patient outcomes and the delivery of integrated, patient-centred musculoskeletal care, particularly within the context of South Africa’s NHI framework.

## Appendix 1

### Participant consent sheet

DEVELOPMENT OF REFERRAL PATHWAYS OF PROGRESSIVE DEGENERATIVE JOINT DISORDERS FOR COMMUNITY PHARMACISTS

1. I have been given a Participant Information Sheet which explains the nature and processes involved in this study, which is attached hereto.
2. I was given time to read it, or had it read to me, in the language I best understand.
3. I was given time to ask any questions I wanted to and found any answers given to me to be reasonable and satisfactory.
4. I believe I fully understand why the study is being conducted and what the intended outcomes will be.
5. I understand that there will be no immediate benefit to me, should I agree to participate, nor will I receive any payment; conversely, participation will not cost me anything but my time.
6. I understand that, even if I initially consent to take part in the study, I may subsequently withdraw at any time and would not be required to give any reasons; if that happened, any data collected about me for the purposes of the study would immediately be destroyed, unless I give consent for it to be retained.
7. I have been given a range of contact details, listed below. If I require further information or become concerned about any aspect of this study, I am free to speak to any of these contacts.

### Contact details

Tumelo Modau, Principal Investigator, by e-mail at 2611806@students.wits.ac.za, Dr Ané Orchard, Supervisor, on telephone no. 011 7 17 2317, or by e-mail at ane.orchard@wits.ac.za and Prof. Demitri Constantinou, Supervisor, on telephone no. 011 717 3396; demitri.constantinou@wits.ac.za.

Professor CB Penny, Chairperson of the Human Research Ethics Committee (Medical) at the University of Witwatersrand, on telephone no. 011 717 2301, or by e-mail at Clement.Penny@wits.ac.za.

Ms. Z Ndlovu or Mr Rhulani Mkansi, Committee Secretariat, telephone nos.: 011 717 2700 or 1234, or by e-mail at: Zanele.Ndlovu@wits.ac.za or Rhulani.Mkansi@wits.ac.za.

Name of Participant: ________________________________________

Date: ________________________________________

Place: ________________________________________

Signature or mark ________________________________________

Witnessed by:

Name of Witness: ________________________________________

Signature: ________________________________________

Date: ________________________________________

## Appendix 2

### Questionnaire for healthcare providers

DEVELOPMENT OF REFERRAL PATHWAYS OF PROGRESSIVE JOINT DISORDERS FOR COMMUNITY PHARMACISTS

Instructions

– Please answer all the questions.
– Tick the relevant box except where specific information is required.

Section A: Demographic information

1. Gender (for statistical purposes only)
  Female: □   Male: □   Other: □   Prefer not to answer: □
2. Race (for statistical purposes only)
  Black: □   White: □   Indian: □   Mixed race:      □   Asian: □
  Other (please specify): _______________   Prefer not to answer: □
3. Age (for statistical purposes only)
  Under 30: □   30–39: □   40–49: □   50–59: □   60 and above: □
4. What is your current profession?
  Physiotherapists: □   Occupational therapists: □   Acupuncturists: □
  Chiropractors: □   Osteopaths: □   Biokineticists: □   Podiatrist: □
  Other (please specify): ___________________     □
5. Are you currently practising in South Africa?
  Yes: □   No: □
6. Which province are you currently working in?
  Eastern Cape: □   Free State: □   Gauteng: □   KwaZulu-Natal: □
  Limpopo: □   Mpumalanga: □   Northern Cape: □   North West: □
  Western Cape: □   Outside South Africa: □
7. How long have you been practising in your field of study (years)?
  Under 2: □   3–5: □   6–10: □   11–20: □   21–30: □   31 and above: □
8. Which institution did you obtain your undergraduate qualification?
  ______________________________

Section B: Practice

Progressive joint disorders are musculoskeletal disorders affecting the joints, whereby there is irreversible loss of articular cartilage, followed by joint pain and dysfunction. They include osteoarthritis, rheumatoid arthritis, psoriatic arthritis, ankylosing spondylitis and gout.

1. Are you familiar with progressive joint disorders?
  Yes: □   No: □   Somewhat: □
2. Which progressive joint disorders do you treat (you may select more than one)?
  Ankylosing spondylitis: □   Gout: □   Psoriatic arthritis: □
  Rheumatoid arthritis: □   Osteoarthritis: □   Other (please specify):_____________□
3. Which progressive joint disorders do you not treat (you may select more than one)?
  Osteoarthritis: □   Other (please specify): ___________________ □
4. What are the reasons for not treating the above selected progressive joint disorders?
  Condition              Reason
  _________________ ___________________________________
  _________________ ___________________________________
  _________________ ___________________________________
5. Is there a guideline(s) that you use when managing patients with progressive joint disorders?
  Yes: □   No: □
  Specify if Yes: __________________________
6. Do you ever recommend any medicinal products for your patients with progressive joint disorders?
  Yes: □   No: □
  Specify if Yes: __________________________
7. Once you realise that a patient with progressive joint disorders is not improving, what is your next step?
  _______________________________
8. Do you receive referrals from any of the following healthcare providers and how often?
  **Table:**
  	At least weekly	At least monthly	A few times a year	Never, but would consider	Never	Unfamiliar
  Pharmacists	□	□	□	□	□	□
  Physiotherapists	□	□	□	□	□	□
  Occupational therapists	□	□	□	□	□	□
  Acupuncturists	□	□	□	□	□	□
  Chiropractors	□	□	□	□	□	□
  Osteopaths	□	□	□	□	□	□
  Biokineticists	□	□	□	□	□	□
  Podiatrist	□	□	□	□	□	□
  Medical doctor	□	□	□	□	□	□
  Other (please specify)	______________________					
9. Have you ever referred any of your patients to any of the following healthcare providers and how often?
  **Table:**
  	At least weekly	At least monthly	A few times a year	Never, but would consider	Never	Unfamiliar
  Pharmacists	□	□	□	□	□	□
  Physiotherapists	□	□	□	□	□	□
  Occupational therapists	□	□	□	□	□	□
  Acupuncturists	□	□	□	□	□	□
  Chiropractors	□	□	□	□	□	□
  Osteopaths	□	□	□	□	□	□
  Biokineticists	□	□	□	□	□	□
  Podiatrist	□	□	□	□	□	□
  Medical doctor	□	□	□	□	□	□
  Other (please specify)	______________________					
10. What is the most common reason for referring your patients to any of the above healthcare providers?
  _______________________________
11. Do you collaborate with any of the following healthcare providers?
  Physiotherapists: □   Occupational therapists: □   Acupuncturists: □
  Chiropractors: □  Osteopaths: □   Biokineticists: □   Podiatrist: □
  Other (please specify): ___________________□
12. Will it be beneficial if pharmacists refer patients to your practice?
  Yes: □   No: □
  Please give reasons __________________________
13. How would you describe the type of patient that should be referred to you by pharmacists?
  __________________________
14. How often do you generally see these patients?
  More than once a week: □   Weekly:    □   Bi-monthly: □
  Monthly: □   Quarterly: □  Other (please specify): ____________□
15. What makes your treatment beneficial for the patients?
  __________________________
16. Do you require a referral letter for new patients?
  Yes: □    No: □
17. Have any of your patients mentioned that they are or have been treated by any of the above healthcare providers?
  Yes: □    No: □
18. If a patient mentions that they are being treated by any other healthcare provider, do you contact the provider?
  Yes: □    No: □
19. Are you willing to engage further through an online discussion/interview regarding this topic?
  Yes: □    No: □
20. Are you willing to contact two of your patients for us to interview regarding their experience?
  Yes: □    No: □

## Appendix 3

**TABLE 1-A3:** Demographic and practice characteristics of participating non-physician healthcare providers.

Characteristics	Categories	Healthcare professional																Total (*n* = 266)	
		Physiotherapists (*n* = 37) (13.9%)		OT (*n* = 48)(18.1%)		Acupuncturist(*n* = 10) (3.8%)		Chiropractor(*n* = 69) (25.9%)		Osteopaths(*n* = 6) (2.3%)		Bio (*n* = 32)(12.0%)		Podiatrist (*n* = 27) (10.2%)		Other (*n* = 37)(13.9%)			
		*n*	%	*n*	%	*n*	%	*n*	%	*n*	%	*n*	%	*n*	%	*n*	%			*n*	%
Gender	Female	23	62.0	45	94.0	6	60	44	63.8	2	33.3	27	84.4	15	55.6	30	81.1	192	72.2
	Male	14	37.8	3	06.3	4	40	25	36.2	4	66.7	5	15.6	12	44.4	7	18.9	74	27.8
Race	Black people	12	32.4	8	16.7	0	0	2	02.9	0	00.0	2	06.2	3	11.1	5	13.5	32	12.1
	White people	15	40.5	34	70.8	6	60	595	85.5	6	100	28	87.5	16	59.3	26	70.3	190	71.4
	Indian people	4	10.8	4	08.3	1	10	6	08.7	0	00.0	0	00.0	6	22.2	4	10.8	25	09.4
	Mixed-race people	6	16.2	2	04.2	1	10	1	01.4	0	00.0	1	03.1	0	00.0	2	05.4	13	04.9
	Other (Asian, prefers not to answer)	0	00.0	0	00.0	2	20	1	01.4	0	00.0	1	03.1	2	07.4	0	00.0	6	02.3
Age group (years)	Below 29	11	29.7	8	16.7	0	0	12	17.4	0	00.0	18	56.3	1	03.7	5	13.5	55	20.7
	30–39	13	35.1	21	43.8	3	30	28	40.6	0	00.0	9	28.1	10	37.0	11	29.7	95	35.7
	40 – 49	7	18.9	6	12.5	3	30	12	17.4	1	16.7	5	15.6	7	25.9	11	29.7	52	19.6
	50–59	3	08.1	9	18.8	3	30	14	20.3	3	50.0	0	00.0	5	18.5	8	21.6	45	16.9
	Over 60	3	08.1	4	08.3	1	10	3	04.3	2	33.3	0	00.0	4	14.8	2	05.4	19	7.1
Years inpractice	Under 5	11	29.7	6	12.5	2	20	16	23.2	0	00.0	18	56.3	2	07.4	8	21.6	63	23.7
	6–10	9	24.3	15	31.3	1	10	22	31.9	1	16.7	5	15.6	6	22.2	4	10.8	63	23.7
	11–20	7	18.9	12	25.0	2	20	9	13.0	1	16.7	7	21.9	7	25.9	14	37.8	59	22.2
	21–30	6	16.2	6	12.5	3	30	19	27.5	3	50.0	2	06.2	8	29.6	8	21.6	55	20.7
	31 and above	4	10.8	9	18.8	2	20	3	04.3	1	16.7	0	00.0	4	14.8	3	08.1	26	9.8
PJD managed	Ankylosing spondylitis	26	70.3	15	31.3	7	70	55	79.7	6	100	18	56.3	11	40.7	17	45.9	155	58.3
	Gout	15	40.5	13	27.1	7	70	34	49.3	5	83.3	10	31.3	26	96.3	31	83.8	141	53.0
	Psoriatic arthritis	10	27.0	12	25.0	3	30	38	55.1	4	66.7	7	21.9	18	66.7	21	56.8	133	50.0
	Rheumatoid arthritis	31	83.8	34	70.8	9	90	57	82.6	6	100	27	84.4	27	100	35	91.9	225	84.6
	Osteoarthritis	34	91.9	34	70.8	10	100	67	97.1	6	100	32	100	26	96.3	35	94.6	244	91.7
	Others	3	08.1	17	35.4	2	20	7	10.1	0	00.0	5	15.6	4	14.8	6	16.2	44	16.5
	Specify†	-	-	-	-	-	-	-	-	-	-	-	-	-	-	-	-	-	-
Recommend medicinal items†	Yes	19	51.4	6	12.5	7	70	51	73.9	3	50.0	9	28.1	15	55.6	32	86.5	142	53.4
	No	18	48.6	42	87.5	3	30	18	26.1	3	50.0	23	71.9	12	44.4	5	13.5	124	46.6
Receive referrals from other HCPs†	Yes	34	91.9	39	81.3	9	90	65	94.2	3	100	28	87.5	26	96.3	30	81.1	237	89.1
	No	3	08.1	9	18.8	1	10	4	05.8	0	00.0	4	12.5	1	03.7	7	18.9	29	10.9
Do you ever refer to other HCPs†	Yes	37	100	44	91.7	9	90	68	98.6	6	100	32	100	27	100	37	100	260	97.74
	No	0	00.0	4	08.3	1	10	1	01.4	0	00.0	0	00.0	0	00.0	0	00.0	6	02.26
Collaborate with other HCPs	Physio	21	56.8	39	81.3	5	50	47	68.1	2	33.3	28	87.5	23	85.2	25	67.6	190	71.43
	OT	23	62.2	20	41.7	3	30	16	23.2	2	33.3	6	18.8	12	44.4	11	29.7	93	35.0
	Acupuncturist	1	02.7	1	02.1	2	20	12	17.4	3	50.0	2	06.3	1	03.7	13	35.1	35	13.2
	Chiro	3	08.1	8	16.7	5	50	28	40.6	1	16.7	15	46.9	16	59.3	23	62.2	99	37.2
	Osteo	3	08.1	1	02.1	2	20	3	04.3	3	50.0	4	12.5	3	11.1	9	24.3	28	10.5
	Bio	18	48.6	15	31.3	5	50	62	89.9	4	66.7	9	28.1	19	70.4	18	48.6	150	56.4
	Podiatrists	15	37.8	7	14.6	2	20	31	44.9	3	50.0	14	43.8	7	25.9	12	32.4	90	33.8
	Pharmacists	15	37.8	1	02.1	1	10	29	42.0	1	16.7	1	03.1	8	29.6	7	18.9	62	23.3
	None	3	08.1	6	12.5	5	50	2	02.9	0	00.0	1	03.1	1	03.7	1	02.7	19	7.1
	Other	10	27.0	8	16.7	4	40	21	30.4	2	33.3	6	18.8	7	25.9	8	21.6	66	24.8
	Specify‡	-	-	-	-	-	-	-	-	-	-	-	-	-	-	-	-	-	-
Which HCPs should pharmacists collaborate with†	Physio	36	97.3	41	85.4	8	80	63	91.3	5	83.3	30	93.8	22	81.5	27	73.0	232	87.2
	OT	26	70.3	41	85.4	6	60	46	66.7	3	50.0	24	75.0	15	55.6	24	64.9	185	70.0
	Acupuncturist	12	32.4	15	31.3	10	10	29	42.0	3	50.0	12	37.5	6	22.2	24	64.9	111	41.74
	Chiro	19	51.4	23	47.9	8	80	67	97.1	4	66.7	18	56.3	14	51.9	26	70.3	179	67.3
	Osteo	17	45.9	20	41.7	7	70	47	68.1	5	83.3	23	71.9	10	37.0	24	64.9	153	57.5
	Bio	20	54.1	26	54.2	7	70	46	66.7	4	66.7	31	96.9	17	63.0	25	67.6	176	66.2
	Podiatrists	18	48.6	27	56.3	5	50	49	71.0	3	50.0	19	59.4	25	92.6	24	64.9	170	63.1
	Only medical doctors	1	02.7	7	14.6	0	0	1	01.4	1	16.7	1	03.1	3	11.1	3	08.1	17	06.4
	None	0	00.0	0	00.0	0	0	1	01.4	0	00.0	0	00.0	0	00.0	0	00.0	1	00.4
Do you require a referral letter†	Yes	15	40.5	30	60.4	3	30	3	04.3	0	00.0	5	15.6	3	11.1	5	13.5	63	23.7
	No	22	59.5	18	39.6	7	70	66	95.7	6	100	27	84.4	24	88.9	33	89.2	203	76.3

Physio, Physiotherapists; OT, Occupational therapists; Chiro, Chiropractors; Osteo, Osteopaths; Bio, Biokineticists; PJD, progressive joint disorders; HCPs, healthcare providers.

† , Physiotherapists – Spondylolisthesis, all muscle, ligament, tendon and joint pathologies, sports injuries. OT – Spine or back and neck–related disorders, fibromyalgia, mobility issues, seropositive arthritis. Acupuncturist – Juvenile idiopathic arthritis, lumbago. Chiropractor – Facet arthrosis, spinal stenosis, disc herniations, osteoporosis, Reiter’s syndrome, spondylolisthesis. Bio – Spondylolisthesis, spondylosis, scoliosis, lumbar disc disease, osteopenia. Podiatrist – Lower limb conditions, ingrown nails, diabetic arthropathy. Other – Systemic lupus erythematosus, bursitis, spondyloarthritis, spondylolisthesis, spondylolysis (degenerative disc disease).

‡ , Physiotherapists – Dieticians, GPs, orthotists, nurses. OT – Orthopaedic surgeons, rheumatologists, GPs, dieticians, psychiatrists, psychologists. Acupuncturist – GPs, Lyno therapists, internists, neurologists, physiatrists, rheumatologists, nephrologists, obstetricians and gynaecologists, psychiatrists, addiction medicine physicians, occupational medicine physicians, family medicine doctors (GPs), endocrinologists. Chiropractor – Fitness trainers, homeopaths, GPs, Orthotists, orthopaedic surgeons, rheumatologists, neurologists, dieticians. Osteopaths – Homeopaths, orthopaedic surgeons. Bio – Homeopaths, dieticians, GPs, orthopaedic surgeons, rheumatologists. Podiatrist – GPs, orthotists, rheumatologists, orthopaedic surgeons, orthopods, vascular surgeons. Other – GPs, rheumatologists, neurologists, physiologists.

## References

[1] Rezuș E, Cardoneanu A, Burlui A, et al. The link between inflammaging and degenerative joint diseases. Int J Mol Sci. 2019;20(3):614. 10.3390/ijms20030614 30708978 PMC6386892

[2] Fu TC, Lane NE, Lee SH, Chen JC, Hsu SF, Chang CM. Editorial: Rehabilitation and alternative medicine in the healthcare for chronic rheumatic pain disorders. Front Med. 2025;12:1586105. 10.3389/fmed.2025.1586105 PMC11968693 40190577

[3] Yoo YM, Kim KH. Facet joint disorders: From diagnosis to treatment. Korean J Pain. 2024;37(1):3–12. 10.3344/kjp.23228 38072795 PMC10764212

[4] Mills GA, Dey D, Kassim M, Yiwere A, Broni K. Diagnostic tool for early detection of rheumatic disorders using machine learning algorithm and predictive models. BioMedInformatics. 2024;4(2):1174–1201. 10.3390/biomedinformatics4020065

[5] Kronbi F, Rkain H, Ez-zaoui S, et al. Attitudes, practices and perceived barriers toward implementing non-pharmacological management for rheumatoid arthritis among rheumatologists: An online cross-sectional survey. Reumatologia. 2024;62(4):250–258. 10.5114/reum/191792 39381732 PMC11457308

[6] Singer BA, Feng J, Chiong-Rivero H. Early use of high-efficacy therapies in multiple sclerosis in the United States: Benefits, barriers, and strategies for encouraging adoption. J Neurol. 2024;271(6):3116–3130. 10.1007/s00415-024-12305-4 38615277 PMC11136864

[7] Shi Y, Wu W. Multimodal non-invasive non-pharmacological therapies for chronic pain: Mechanisms and progress. BMC Med. 2023;21(1):372. 10.1186/s12916-023-03076-2 37775758 PMC10542257

[8] Huijbrechts EJ, Dekker J, Tenten-Diepenmaat M, Gerritsen M, Van der Leeden M. Clinical guidance for podiatrists in the management of foot problems in rheumatic disorders: Evaluation of an educational programme for podiatrists using a mixed methods design. J Foot Ankle Res. 2021;14(1):15. 10.1186/s13047-020-00435-7 33632287 PMC7908782

[9] Shahid A, Inam-Ur-Raheem M, Iahtisham-Ul-Haq, et al. Diet and lifestyle modifications: An update on non-pharmacological approach in the management of osteoarthritis. J Food Process Preserv. 2022;46(8):e16786. 10.1111/jfpp.16786

[10] Loubani K, Polo KM, Baxter MF, Rand D. Identifying facilitators of and barriers to referrals to occupational therapy services by Israeli cancer health care professionals: A qualitative study. Am J Occup Ther. 2024;78(1):7801205050. 10.5014/ajot.2024.050414 38224354

[11] Babatunde OO, Adetunji O, Alonge I, et al. Process and feasibility of implementing guideline recommendations for the care of osteoarthritis in West Africa. BMJ Glob Health. 2025;10(6):e018714. 10.1136/bmjgh-2024-018714 PMC12182005 40537272

[12] Chavan, AA, Kumbhar, SB, Shinde, VR, et al. Role of pharmacist in healthcare system. GSC Biol Pharm Sci. 2023;24(1):36–45. 10.30574/gscbps.2023.24.1.0261

[13] Dineen-Griffin S, Benrimoj SI, Garcia-Cardenas V. Primary health care policy and vision for community pharmacy and pharmacists in Australia. Pharm Pract (Granada). 2020;18(2): 1967. 10.18549/PharmPract.2020.2.1967 32477437 PMC7243858

[14] Lott BE, Anderson EJ, Villa Zapata L, et al. Expanding pharmacists’ roles: Pharmacists’ perspectives on barriers and facilitators to collaborative practice. J Am Pharm Assoc (2003). 2021;61(2):213–220.e1. 10.1016/j.japh.2020.11.024 33359117

[15] Hooper AD, Marquez J, Bajorek B, Cooper J, Newby D. Understanding pharmacists’ engagement in sport and exercise medicine, including pharmacist-physiotherapist collaboration: A qualitative study and COM-B analysis. Explor Res Clin Soc Pharm. 2025;18:100593. 10.1016/j.rcsop.2025.100593 40212890 PMC11984990

[16] Sun Q, Wan C, Xu Z, Huang Y, Xi X. Association of pharmaceutical care barriers and role ambiguity and role conflict of clinical pharmacists. Front Pharmacol. 2023;14:1103255. 10.3389/fphar.2023.1103255 37229262 PMC10203618

[17] Naidoo V, Moodley R, Bangalee V, Suleman F. New medicine service by community pharmacists: An opportunity to enhance universal health coverage at a primary health level in South Africa. Inquiry. 2023;60:469580221146834. 10.1177/00469580221146834 36625010 PMC9834920

[18] Li M, Raven J, Liu X. Feminization of the health workforce in China: Exploring gendered composition from 2002 to 2020. Hum Resour Health. 2024;22(1):15. 10.1186/s12960-024-00898-w 38373975 PMC10877893

[19] Matseke MG. Taking stock of the healthcare workforce in the public health sector of South Africa during COVID-19: Implications for future pandemics. Afr J Public Sect Dev Gov. 2023;6(1):59–76. 10.55390/ajpsdg.2023.6.1.5

[20] De Villiers K. Bridging the health inequality gap: An examination of South Africa’s social innovation in health landscape. Infect Dis Poverty. 2021;10(1):19. 10.1186/s40249-021-00804-9 33648585 PMC7919075

[21] Krebs F, Lorenz L, Nawabi F, et al. Recruitment in health services research – A study on facilitators and barriers for the recruitment of community-based healthcare providers. Int J Environ Res Public Health. 2021;18(19):10521. 10.3390/ijerph181910521 34639820 PMC8508262

[22] AHPCSA. Registers. The allied health professions council of South Africa [homepage on the Internet]. 2025 [cited 2025 Aug 09]. Available from: https://ahpcsa.co.za/practitioners/

[23] HPCSA. HPCSA statistics [homepage on the Internet]. 2025 [cited 2025 Aug 09]. Available from: https://www.hpcsa.co.za/statistics

[24] Tang S, Zhang C, Oo WM, et al. Osteoarthritis. Nat Rev Dis Primers. 2025;11(1):10. 10.1038/s41572-025-00594-6 39948092

[25] Gilchrist R, Kholvadia A. Team approach to osteoarthritis management: Viewpoints of biokineticists and physiotherapists in South Africa. S Afr J Sports Med. 2023;35(1):v35i1a15260. 10.17159/2078-516X/2023/v35i1a15260 PMC10798614 38249754

[26] Ramos-Petersen L, Reinoso-Cobo A, Ortega-Avila AB, et al. A clinical practice guideline for the management of the foot and ankle in rheumatoid arthritis. Rheumatol Int. 2024;44(8):1381–1393. 10.1007/s00296-024-05633-1 38850327 PMC11222212

[27] Roberts A, Harris K, Outen B, et al. Osteopathic manipulative medicine: A brief review of the hands-on treatment approaches and their therapeutic uses. Medicines (Basel). 2022;9(5):33. 10.3390/medicines9050033 35622072 PMC9143587

[28] Gioglou G, Trevlaki E, Trevlakis E. Physical therapy approaches in ankylosing spondylitis: A systematic review. Int J Curr Sci Res Rev. 2023;6(2):1629–1637. 10.47191/ijcsrr/V6-i2-83

[29] Zhu Q, Chen J, Xiong J, et al. The efficacy of moxibustion and acupuncture therapy for ankylosing spondylitis: A protocol for an overview of systematic reviews and meta-analysis. Medicine (Baltimore). 2021;100(15):e25179. 10.1097/MD.0000000000025179 33847616 PMC8051999

[30] Yong CY, Hamilton J, Benepal J, et al. Awareness of axial spondyloarthritis among chiropractors and osteopaths: Findings from a UK Web-based survey. Rheumatol Adv Pract. 2019;3(2):rkz034. 10.1093/rap/rkz034 31616854 PMC6785804

[31] Schwendner M, Seule M, Meyer B, Krieg SM. Management of spine fractures in ankylosing spondylitis and diffuse idiopathic skeletal hyperostosis: A challenge. Neurosurg Focus. 2021;51(4):E2. 10.3171/2021.7.FOCUS21330 34598125

[32] Silva ME, De Melo EBB, Cabral MAL, et al. Effects of acupuncture on the signs and symptoms of people with rheumatic diseases: A scoping review. Complement Ther Med. 2025;91:103183. 10.1016/j.ctim.2025.103183 40274134

[33] Wojtania J, Płeska K, Łepik M, et al. Gout and its impact on physical activity. Qual Sport. 2024;21:51463. 10.12775/QS.2024.21.51463

[34] McIntyre A, Mackenzie L, Harvey M. Engagement of general practitioners in falls prevention and referral to occupational therapists. Br J Occup Ther. 2019;82(2):71–79. 10.1177/0308022618804752

[35] Gassara Z, Feki A, Hakim Z, et al. Foot involvement in psoriatic arthritis: Prevalence, clinical and radiological features. Foot Ankle Surg. 2024;30(6):465–470. 10.1016/j.fas.2024.03.006 38538387

[36] Sandoval DC, Fernández-Ávila DG. Assessment tools in psoriatic arthritis: A review. Rev Colomb Reumatol (Engl Ed). 2023;30:S75–S86. 10.1016/j.rcreue.2022.11.003

[37] Overcash MD, Chillura C, Fender SP, et al. Psoriatic arthritis: The role of the nonphysician clinician in the diagnosis and treatment of patients with psoriasis. Drugs Ther Perspect. 2021;37(4):162–174. 10.1007/s40267-021-00814-5

[38] Romeyke T, Stummer H. Multimodal approaches in the treatment of chronic peripheral neuropathy – Evidence from Germany. Int J Environ Res Public Health. 2024;21(1):66. 10.3390/ijerph21010066 38248531 PMC10815843

[39] Rzeczycki P, Rasner C, Lammlin L, et al. Cannabinoid receptor type 2 is upregulated in synovium following joint injury and mediates anti-inflammatory effects in synovial fibroblasts and macrophages. Osteoarthr Cartil. 2021;29(12):1720–1731. 10.1016/j.joca.2021.09.003 PMC8883578 34537380

[40] Kriel C, Weilbach JT, Caldwell LL. Leisure education and recreation participation: A niche for recreational therapy in South Africa. World Leis J. 2022;64(4):399–415. 10.1080/16078055.2022.2058995

[41] Graham K, Matricciani L, Banwell H, et al. Australian podiatrists scheduled medicine prescribing practices and barriers and facilitators to endorsement: A cross-sectional survey. J Foot Ankle Res. 2022;15(1):11. 10.1186/s13047-022-00515-w 35135610 PMC8822637

[42] Lyhnebeck AB, Sahl Andersen J, Skou ST, Risør MB, Guassora AD. Trust, humor, and the balance of involvement – Patients with musculoskeletal conditions and comorbidities and their expectations towards physiotherapists. J Multimorb Comorb. 2025;15:26335565251321919. 10.1177/26335565251321919 40144307 PMC11938482

[43] Oğuz F, Arslan M. Knowledge and behavior of community pharmacists towards detecting drug-drug interactions. Eur J Life Sci. 2023;2(1):39–44. 10.55971/EJLS.1266042

[44] Kc B, Alrasheedy AA, Mohamed Ibrahim MI, et al. Combatting opioid misuse, overuse and abuse: A systematic review of pharmacists’ services and outcomes. Pain Manag. 2024;14(9):519–529. 10.1080/17581869.2024.2411930 39439259 PMC11728331

[45] Chen J, Ma L, Li X, et al. Relation labeling in product knowledge graphs with large language models for e-commerce. Int J Mach Learn Cyber. 2024;15(12):5725–5743. 10.1007/s13042-024-02274-5

[46] Vadivel S, Mani P, Anezi NA, et al. Knowledge and awareness about occupational therapy among healthcare professionals in Al-Ahsa. World J Biol Pharm Health Sci. 2021;8(1):8–12. 10.30574/wjbphs.2021.8.1.0104

[47] Engel RM, Beirman R, Grace S. An indication of current views of Australian general practitioners towards chiropractic and osteopathy: A cross-sectional study. Chiropr Man Therap. 2016;24:37. 10.1186/s12998-016-0119-6 PMC5088656 27822360

[48] Menz HB, Harrison C, Bayram C. Characteristics of general practitioner referrals to podiatrists in Australia, 2000–2016. Public Health. 2021;193:10–16. 10.1016/j.puhe.2021.01.015 33677392

[49] Toloui-Wallace J, Forbes R, Thomson OP, Costa N. Fluid professional boundaries: Ethnographic observations of co-located chiropractors, osteopaths and physiotherapists. BMC Health Serv Res. 2024;24(1):344. 10.1186/s12913-024-10738-1 38491351 PMC10943826

[50] Rathore FA, Anwar F, Younas U. Multidisciplinary team working in rehabilitation medicine: Advantages and challenges. J Pak Med Assoc. 2024;74(2):409–412. 10.47391/JPMA.24-13 38419251

[51] Wilbur K, Kelly D, Jorgenson D. Interprofessional collaboration in pharmacist-led primary care clinics. Can Pharm J (Ott). 2025;158(3):172–179. 10.1177/17151635241312423 40093505 PMC11907563

[52] Mbata AO, Ogbewele EG, Nwosu NT. Pharmacists in global primary healthcare systems: A comprehensive model for community health empowerment. Int J Front Med Surg Res. 2024;6(2):19–28. 10.53294/ijfmsr.2024.6.2.0044

[53] Goodman SB, Jiranek W, Petrow E, Yasko AW. The effects of medications on bone. J Am Acad Orthop Surg. 2007;15(8):450–460. 10.5435/00124635-200708000-00002 17664365

[54] Altuwayrib S, Win KT, Freeman M. Medication adherence support applications for chronic arthritis patients: Healthcare providers’ perspective in Saudi Arabia. Stud Health Technol Inform. 2024;310:1056–1060. 10.3233/SHTI231126 38269976

[55] Ngusie HS, Ahmed MH, Mengiste SA, et al. The effect of capacity building evidence-based medicine training on its implementation among healthcare professionals in Southwest Ethiopia: A controlled quasi-experimental outcome evaluation. BMC Med Inform Decis Mak. 2023;23(1):172. 10.1186/s12911-023-02272-7 37653419 PMC10472735

[56] Harvey-Sullivan A, Lynch H, Tolley A, Gitlin-Leigh G, Kuhn I, Ford JA. What impact do self-referral and direct access pathways for patients have on health inequalities? Health Policy. 2024;139:104951. 10.1016/j.healthpol.2023.104951 38096622

